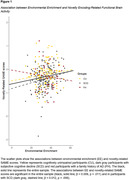# Association between long‐term Environmental Enrichment and Memory‐Related Functional Brain Activity in the DELCODE cohort

**DOI:** 10.1002/alz.088136

**Published:** 2025-01-09

**Authors:** Simon Hass, Joram Soch, Jasmin Kizilirmak, Maxie Liebscher, Emrah Düzel, Klaus Fliessbach, Frank Jessen, Christoph Laske, Oliver Peters, Josef Priller, Sandra Roeske, Anja Schneider, Hartmut Schuetze, Annika Spottke, Stefan Teipel, Michael Wagner, Jens Wiltfang, Franka Gloeckner, Björn H. Schott, Miranka Wirth, Olga M. Klimecki

**Affiliations:** ^1^ German Center for Neurodegenerative Diseases (DZNE), Dresden Germany; ^2^ German Center for Neurodegenerative Diseases (DZNE), Goettingen Germany; ^3^ German Center for Neurodegenerative Diseases (DZNE), Magdeburg Germany; ^4^ Institute of Cognitive Neurology and Dementia Research (IKND), Otto‐von‐Guericke University, Magdeburg Germany; ^5^ University of Bonn Medical Center, Dept. of Neurodegenerative Disease and Geriatric Psychiatry/Psychiatry, Venusberg‐Campus 1, 53127 Bonn, Germany, Bonn Germany; ^6^ German Center for Neurodegenerative Diseases (DZNE), Bonn Germany; ^7^ Excellence Cluster on Cellular Stress Responses in Aging‐Associated Diseases (CECAD), University of Cologne, Cologne Germany; ^8^ Department of Psychiatry, University of Cologne, Medical Faculty, Cologne Germany; ^9^ German Center for Neurodegenerative Diseases (DZNE), Tuebingen Germany; ^10^ Section for Dementia Research, Hertie Institute for Clinical Brain Research and Department of Psychiatry and Psychotherapy, University of Tuebingen, Tuebingen Germany; ^11^ Charité – Universitätsmedizin Berlin, corporate member of Freie Universität Berlin and Humboldt‐Universität zu Berlin – Institute of Psychiatry and Psychotherapy, Berlin Germany; ^12^ German Center for Neurodegenerative Diseases (DZNE), Berlin Germany; ^13^ School of Medicine, Technical University of Munich; Department of Psychiatry and Psychotherapy, Munich Germany; ^14^ University of Edinburgh and UK DRI, Edinburgh United Kingdom; ^15^ Department of Psychiatry and Psychotherapy, Charité, Charitéplatz 1, Berlin Germany; ^16^ Department of Neurology, University of Bonn, Bonn Germany; ^17^ German Center for Neurodegenerative Diseases (DZNE), Rostock Germany; ^18^ Department of Psychosomatic Medicine, Rostock University Medical Center, Rostock Germany; ^19^ Department of Psychiatry and Psychotherapy, University Medical Center, University of Goettingen, Goettingen Germany; ^20^ Neurosciences and Signaling Group, Institute of Biomedicine (iBiMED), Department of Medical Sciences, University of Aveiro, Aveiro Portugal; ^21^ Department of Lifespan Developmental Neuroscience, Faculty of Psychology, Technical University of Dresden, Dresden Germany; ^22^ Department of Psychiatry and Psychotherapy, University Medical Center Goettingen (UMG), Göttingen Germany

## Abstract

**Background:**

Environmental factors account for a considerable percentage of dementia cases. Studies in animal models have shown that environmental enrichment (EE; i.e., stimuli‐rich housing conditions) has positive effects on brain structure, including the memory system. In humans, EE as measured by the engagement in a variety of leisure activities has been associated with better fornix structure and memory (Klimecki et al., 2023). We assessed whether long‐term EE (in terms of engagement in diverse leisure activities) is related to functional brain activity in the memory system of older adults.

**Methods:**

We operationalized individual EE in 372 older participants aged between 60 and 87 years of the DZNE Longitudinal Study on Cognitive Impairment and Dementia (DELCODE) study. We used subscales of the Lifetime of Experiences Questionnaire (LEQ; Valenzuela & Sachdev, 2007) that capture the frequency of engagement in diverse leisure activities in young adulthood and middle life (13‐30 and 30‐65 years). Memory‐related brain activity was assessed using individual FADE‐SAME scores in a functional magnetic resonance imaging (fMRI) paradigm on visual memory encoding and recognition. The scores capture the similarity of older adults’ brain activity patterns with typical activations of younger adults’ (Soch et al., 2021). We performed multiple regression analyses between long‐term EE as independent variable and FADE‐SAME scores related to novelty processing and subsequent memory as dependent variables.

**Result:**

Long‐term EE was significantly associated with novelty‐based SAME scores. More specifically, older participants with higher EE in early and middle life showed a higher similarity of functional brain activity patterns during novelty processing with the patterns seen in younger adults (see Figure 1). Exploratory subgroup analysis showed that this association was predominantly found in participants with Subjective Cognitive Decline (SCD, n = 199). No other significant findings were obtained.

**Conclusion:**

Engagement in a variety of leisure activities during early and middle life is related to more “youth‐like” memory‐related brain activity patterns in older adults, including older individuals at increased risk of AD. Higher EE during early life might contribute to preservation or promotion of memory functions in later life.